# Genetically Predicted Atrial Fibrillation and Valvular Heart Disease: A Two-Sample Mendelian Randomization Study

**DOI:** 10.3389/fcvm.2022.845734

**Published:** 2022-03-28

**Authors:** Jie Gao, Yulin Bai, Hongwen Ji

**Affiliations:** ^1^Department of Anesthesiology, Fuwai Hospital, Chinese Academy of Medical Science and Peking Union Medical College, Beijing, China; ^2^Department of Cardiac Surgery, The 7th People's Hospital of Zhengzhou, Zhengzhou, China

**Keywords:** atrial fibrillation (AF), valvular heart disease (VHD), genome-wide association study (GWAS), Mendelian randomization (MR), risk

## Abstract

**Background:**

Previous studies have found atrial fibrillation (AF) is associated with valvular heart disease (VHD). However, whether there is a causal relationship between these two diseases or it is just a result of bias caused by confounding factors is uncertain. This study aims to examine the potential causal association between AF and VHD by using Mendelian randomization.

**Methods:**

In order to examine the causal relationship between AF and VHD, we performed a two-sample Mendelian randomization study by collecting exposure and outcome data from genome-wide association study (GWAS) datasets. We utilized data from FinnGen project (FinnGen, 11,258 cases for VHD including rheumatic fever, 3,108 cases for non-rheumatic VHD, and 75,137 cases for participants) and European Bio-informatics Institute database (EBI, 55,114 cases for AF and 482,295 cases for participants). Inverse-variance weighted (IVW), MR-Egger, and weighted median approaches were performed to estimate the causal effect.

**Results:**

The Mendelian randomization analysis indicated that AF increased the risk of VHD by all three MR methods [For VHD including rheumatic fever: IVW, odds ratio (OR) = 1.255; 95% confidence interval (CI), 1.191~1.322; *p* = 1.23 × 10^−17^; Weighted median, OR = 1.305, 95% CI, 1.216~1.400, *p* = 1.57 × 10^−13^; MR-Egger, OR = 1.250, 95% CI, 1.137~1.375, *p* = 1.69 × 10^−5^; For non-rheumatic VHD: IVW, OR = 1.267; 95% CI, 1.169~1.372; *p* = 6.73 × 10^−9^; Weighted median, OR = 1.400; 95% CI, 1.232~1.591; *p* = 2.40 × 10^−7^; MR-Egger, OR = 1.308; 95% CI, 1.131~1.513; *p* = 5.34 × 10^−4^]. After the one outlier SNP was removed by heterogeneity test, the results remained the same. No horizontal pleiotropic effects were observed between AF and VHD.

**Conclusions:**

Our study provides strong evidence of a causal relationship between AF and VHD. Early intervention for AF patients may reduce the risk of developing into VHD.

## Introduction

Atrial fibrillation (AF) is the most common sustained cardiac arrhythmia. It affects about 10% of the general population (1). About 70% of AF is asymptomatic or present with palpitations, dizziness, and difficulty breathing during mild physical activity (2). AF can lower ejection fraction and produce a variety of side effects, including tachycardia, thromboembolism, and a 15 20% reduction in cardiac output due to a loss of ventricular filling. Although AF can be treated with drugs and interventional surgery, there are still some patients with AF who tend to have severe complications such as stroke and valvular heart disease (VHD), especially in those with non-valvular AF patients (3, 4).

To date, several observational studies found that significant functional VHD is common in patients with AF, accounting for about 20-27.7%, and this rate is even higher in patients with long-standing AF (5–9). Compared with isolated AF patients, AF with concomitant VHD patients had a 23-55% increased mortality (10–12). Therefore, clarifying the relationship between these two diseases may be a key strategy to reducing patient mortality.

However, due to the limitation of the observational study that it can be easily affected by confounding factors such as environment and selection bias, we cannot get an accurate conclusion. A previous mendelian randomization (MR) analysis suggested a genetic association between AF on various cardiovascular diseases such as stroke and ischemic heart disease (13). But there is no sufficient statistical power to identify the causal relationship between AF and VHD alone at conventional significance thresholds.

To address the gap, we assessed the causal role by using two-sample mendelian randomization (MR) analysis to assess whether a genetically higher risk of VHD is associated with AF. Two-sample MR is a method to assess the causal effect of an exposure on an outcome using an instrument which is defined by one or more single-nucleotide polymorphisms (SNPs), as a proxy for the exposure (14). It is important to find out the association between AF and VHD in order to add insight into the underlying etiology because AF is preventable and treatable.

## Materials and Methods

### Overall Study Design

**Figure 1:**
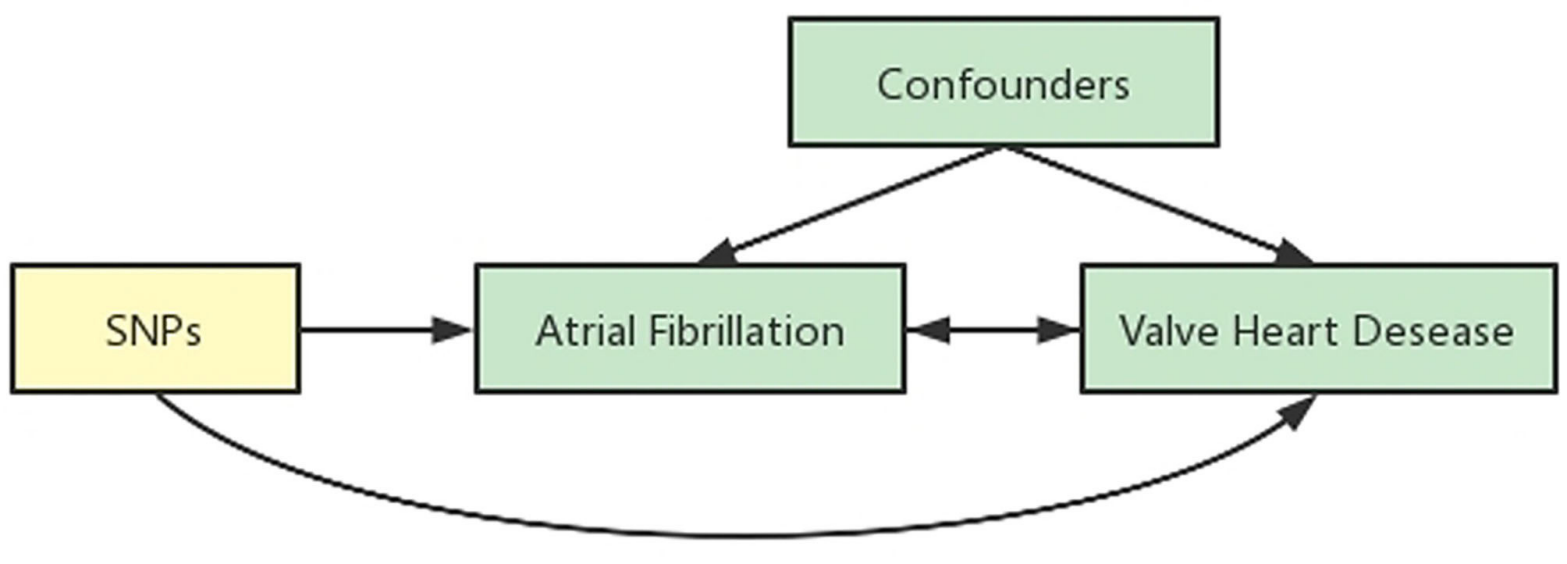
Schematic representation of an MR analysis. We selected SNPs which associated with AF from the European Bio-informatics Institute database and the corresponding effect for these SNPs was estimated based on the risk of VHD obtained from the FinnGen. Because of the randomization and independence of alleles at meiosis, MR is a powerfully predictive tool to assess causal relationships with on bias inherent to observational study designs.

We obtained the summary data from published studies, which have already been approved by institutional review committees in their respective studies. Therefore, no further sanction was required. We used two-sample MR (15, 16) to assess the causal effect between AF and VHD (Figure 1).

### Data Sources

#### Identification of SNPs Associated With AF

Summary statistics of AF were obtained from the public genome-wide association study (GWAS) that had been assembled in the European Bio-informatics Institute (EBI) database at https://gwas.mrcieu.ac.uk/datasets/ebi-a-GCST006061/. Only European ancestry populations were used in our study, which include 55,114 cases and 482,295 control participants.

Genetic variants that passed uncorrelated (r^2^ < 0.001) SNPs associated with the risk factor at thresholds for a genome-wide level of statistical significance (*p* < 5 × 10^−8^) were selected as instruments.

#### Study Outcome: VHD

Data on VHD were drawn from the GWAS summary data sources on the FennGenn database, which is available at https://gwas.mrcieu.ac.uk/datasets/finn-a-I9_VHD/ (VHD including rheumatic fever, 11,258 cases, 75,137 participants) and https://gwas.mrcieu.ac.uk/datasets/finn-a-I9_NONRHEVALV/ (Non-rheumatic VHD, 3,108 cases, 75,137 participants). As described earlier, independent variants that meet the criteria (r^2^ < 0.001, *p* < 5 × 10^−8^) were considered as low correlation.

#### Statistical Analysis

Due to the lack of individual-level GWAS data, we chose to use the recently developed method of MR analysis to evaluate whether there is a causal relationship between AF on VHD.

As a genetic method, MR can enhance inferences about the causal nature of exposure-outcome associations by reducing the likelihood of confounding and eliminating reverse causality in conventional observational studies (17). This is because the genetic alleles associated with exposure are conceptually randomly classified and therefore independent of confounding factors such as self-selected lifestyle and environmental factors, also are not affected by disease, and the time sequence is reasonable. Therefore, genotype can be used as an instrumental variable to infer the association between exposure and disease.

But the outcome can be influenced by the genetic variant through a pathway rather than the exposure alone, known as horizontal pleiotropy, which violates the assumption of MR and can bias causal estimates. To correct for this, Inverse-variance weighted (IVW), MR-Egger, and weighted median analytical approaches were used in our MR analysis. IVW is the most preferred and commonly used statistical method, but it only gives consistent estimates if all of the genetic variants in the analysis are valid instrumental variables. MR-Egger statistical method allows for horizontal pleiotropy in the included instrumental SNPs. Weighted median statistical method is complementary to the MR-Egger regression method. It can provide a valid estimate if there are more than 50% of the information comes from SNPs that are valid instrumental variables. All three methods are based on different horizontal pleiotropic models (18). The value of comparing all the results is that the consistency of the different kinds of methods makes our results more reliable (19, 20).

The statistical coding and related data of this study can be obtained from the corresponding author if you need based on reasonable request. MR analysis were conducted in R, version 4.0.3 (http://www.r-project.org) using the TwoSampleMR package (21, 22).

## Results

### Genetic Instrumental Variables for AF

Identified from previous GWAS database, we find that there are 77 SNPs associated with AF at the genome-wide significance level (*p* < 5 × 10^−8^), as shown in Supplementary Table 1.

**Figure 2:**
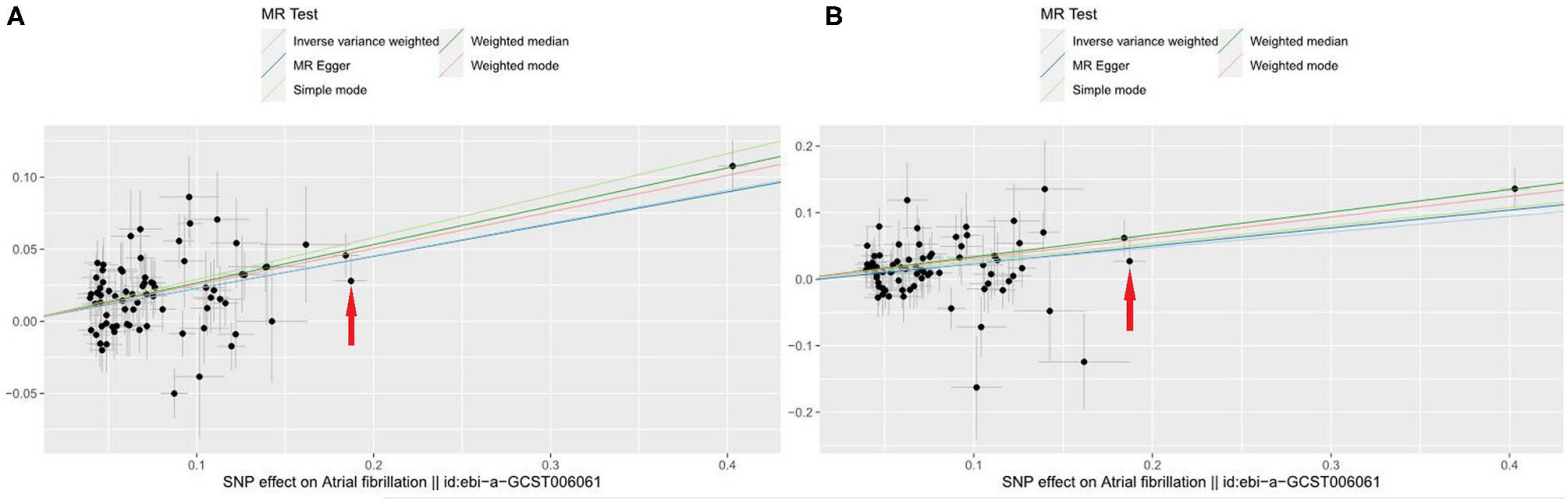
Scatter plot to visualize causal effect of AF on total VHD risk. **(A)** AF on VHD including rheumatic fever; **(B)** AF on non-rheumatic VHD. The slope of the straight line indicates the magnitude of the causal association. Red arrows indicates a certain SNP (rs2106261) (X-axis: beta_exposure = 0.1872; Y-axis: beta_outcome = 0.0280). IVW indicates inverse-variance weighted; and MR, Mendelian randomization.

Of all 77 genetic instruments, an SNP rs2106261 in zinc finger homeobox 3 (ZFHX3) gene locus has previously been reported to have a significant association with AF in European ancestry populations by affecting the myocardial tissue function, regulating myogenic and neuronal differentiation (23, 24). The associations of VHD risk for each genetic variant are shown in Figure 2 and Supplementary Figure 1. We use beta coefficient to describe the correlation coefficient (for Figure 2A, beta = 0.269; for Figure 2B, beta = 0.223).

### Mendelian Randomization Analysis for VHD

**Figure 3:**
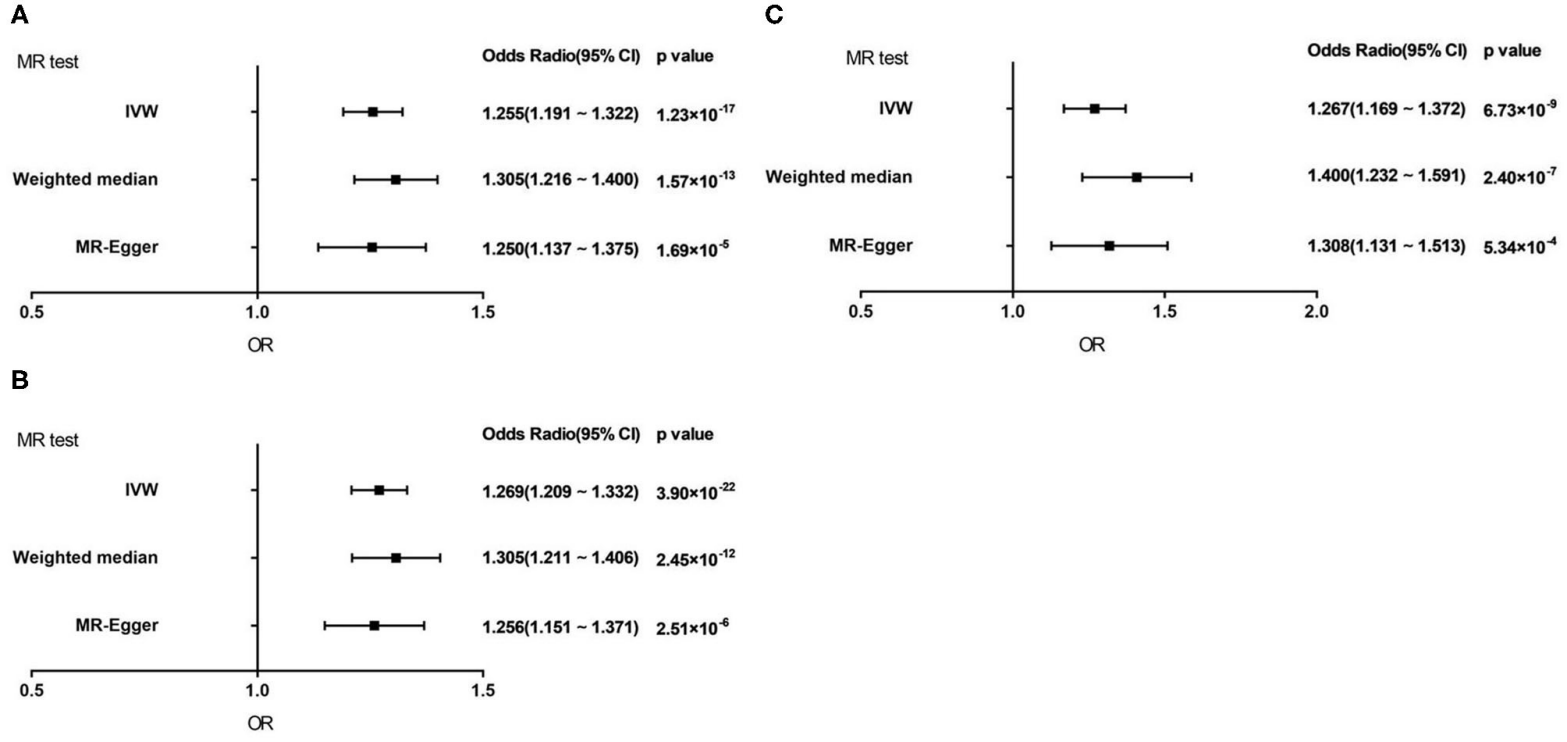
Forest plot to visualize causal effect of AF on total VHD risk by three methods. **(A)** VHD including rheumatic fever. **(B)** VHD including rheumatic fever with 1 SNP excluded. **(C)** Non-rheumatic VHD. IVW, inverse-variance weighted; MR, Mendelian randomization.

Genetically predicted AF showed a consistent association with VHD including rheumatic fever under the IVW method [odds ratio (OR) = 1.255; 95% confidence interval (CI), 1.191 1.322; *p* = 1.23 × 10^−17^]. Similar results were obtained of the weighted median method and MR-Egger method (Weighted median, OR = 1.305; 95% CI, 1.216 1.400; *p* = 1.57 × 10^−13^; MR-Egger, OR = 1.250; 95% CI, 1.137 1.375; *p* = 1.69 × 10^−5^) (Figure 3A).

As for non-rheumatic VHD, the results demonstrated the same trends as the above studies in three statistic methods (IVW, OR = 1.267; 95% CI, 1.169 1.372; *p* = 6.73 × 10^−9^; Weighted median, OR = 1.400; 95% CI, 1.232 1.591; *p* = 2.40 × 10^−7^; MR-Egger, OR = 1.308; 95% CI, 1.131 1.513; *p* = 5.34 × 10^−4^) (Figure 3C).

### Sensitivity Analysis

**1. Heterogeneity Analysis**

Q statistic was conducted to detect heterogeneous outcomes. We found that for VHD including rheumatic fever, there was heterogeneity among 77 SNPs (IVW: Q = 103.277, *p* = 0.017; MR-Egger: Q = 103.290, *p* = 0.020). To identify the specific SNPs responsible for the heterogeneous results, we performed the MR-PRESSO outliner test and found that the heterogeneous results were due to a certain SNP (rs34750263). Based on the three assumptions of two-sample MR analysis, we reanalyzed the association between AF and VHD including rheumatic fever by excluding this outliner SNP, leaving 76 SNPs as genetic instruments. The result showed that after excluding 1 SNPs, the Q statistic revealed no notable heterogeneity under the IVW model and MR-Egger model (IVW: Q = 86.335, *p* = 0.175; MR-Egger: Q = 86.246, *p* = 0.156).

After excluding one outliner SNP, we repeated the analysis to estimate the causal relationships between AF and VHD including rheumatic fever and the result did not change substantially (IVW, OR = 1.269; 95% CI, 1.209 1.332; *p* = 3.90 × 10^−22^; Weighted median, OR = 1.305; 95% CI, 1.211 1.406; *p* = 2.45 × 10^−12^; MR-Egger, OR = 1.256; 95% CI, 1.151 1.371; *p* = 2.51 × 10^−6^; Figure 3B). It suggested that our result is reliable and stable.

**2. Horizontal Pleiotropy Analysis**

**Figure 4:**
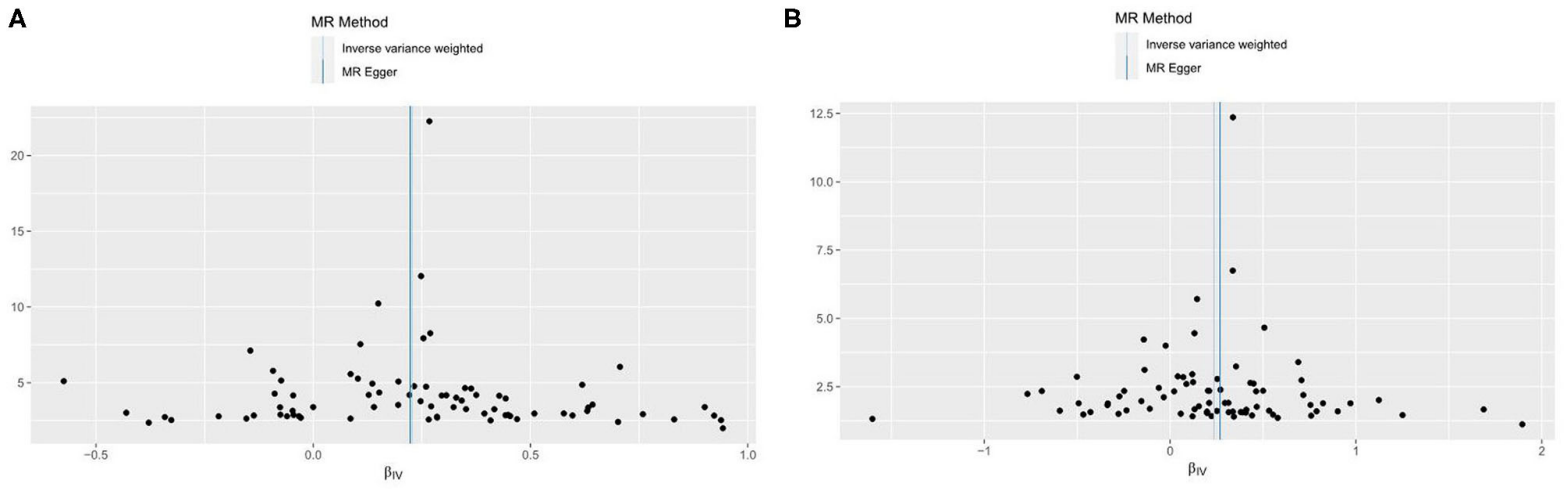
Funnel plots to visualize overall heterogeneity of MR estimates for the effect of AF on VHD. **(A)** VHD including rheumatic fever. **(B)** non-rheumatic VHD. IVW, inverse-variance weighted; MR, Mendelian randomization.

To reduce the bias caused by horizontal pleiotropy, we performed the MR-Egger intercept test, and the visualized results were displayed in funnel plot (Figure 4). The result from the test did not reveal any horizontal pleiotropy in our study (for VHD including rheumatic fever: intercept = 0.0004, *p* = 0.923; for non-rheumatic VHD, intercept = −0.003, *p* = 0.605).

**3. Effects of Individual Genetic Instruments in Relation to VHD**

To verify the influence of each SNP on the overall causal estimate, leave-one-out analysis was performed. No substantial difference appeared in estimated causal effect when systematically removing individual SNP and repeating the MR analysis (Supplementary Figure 2). Therefore, not any single genetic instrument resulted in the estimated effects.

## Discussion

Based on previous observational research, the present study systematically evaluated the association between AF and VHD by MR method, which is a natural RCT, using publicly available large-scale GWAS datasets. With 77 SNPs that were significantly associated with AF as the main instruments in the two-sample MR analysis, we found that to some extent, a genetic predisposition of people with AF can increase the incidence of both VHD including rheumatic fever and non-rheumatic VHD.

VHD is mainly manifested as valve stenosis or insufficiency, which is the main cause of impaired activity tolerance, heart failure, and death in patients (11). According to epidemiological statistics in British, at least 50% of people over the age of 65 suffered from VHD (25). VHD is mainly caused by congenital dysplasia or other pathological changes, such as degenerative diseases, infections, and trauma. Previous studies found that individuals with a diagnosis of coronary heart disease, hypertension, chronic kidney disease and AF had a higher risk of VHD, which has been reported in many large sample-size researches (25, 26). The incidence of VHD also increases with age (25). Recently, the *2021 ESC/EACTS Guidelines for the Management of VHD* (27) suggested that VHD may arise as a consequence of atrial enlargement and mitral annular dilatation in patients with longstanding AF, which means that people have begun to pay attention to the mechanism of secondary valvular disease. Notably, in a study of 47 AF patients, Oren et al. (28) found that people who were diagnosed with permanent lone AF had a 6.5-fold higher likelihood of having tricuspid regurgitation (*p* = 0.0031) and were marginally more likely to have mitral regurgitation compared to those with paroxysmal AF. Utsunomiya et al. (29) reported that functional tricuspid regurgitation with a structurally normal tricuspid valve may occur secondary to chronic AF, which indicated that there might be a relationship between AF and VHD. These results provided a basis for our study.

However, the causal association between these two diseases is not clear yet. Previous genetic studies of VHD mainly focused on genetic risk factors involved in blood pressure or obesity, including high LDL-cholesterol and Lipoprotein(a) (30–33). However, there is no research studied the causal relationship between AF and VHD before. We therefore aimed at filling this research gap.

To systematically examine if VHD is the cause or consequence of AF in the European ancestry population, two-sample MR method was used. In our study, with 77 SNPs that were significantly associated with AF as genetic instruments, we found that 1-SD increase of AF was associated with a 30.5% increase of VHD including rheumatic fever and a 26.7% increase of non-rheumatic VHD. This may be because that the AF and VHD share the same genetic susceptibility factors.

Of the 77 SNPs, only rs2106261, in the transcription factor gene ZFHX3, had previously been reported. Lubitz et al. (34) identified an AF susceptibility gene, the transcription factor ZFHX3, which was originally identified in the Caucasian population by scanning SNPs rs2106261. This finding was subsequently replicated in other populations, such as the Chinese Han population (35) and the Japanese population (36). A recent study published by Zaw et al. (36) showed a similar result with the above researches that the A allele of the rs2106261 SNP was significantly associated with AF, after adjusting for age, sex, diabetes, hypertension, and smoking, which indicated that this SNP variant might be an independent risk marker for AF. ZFHX3 is a candidate tumor suppressor gene for prostate, breast and gastric cancer, which acts by inducing cell cycle arrest. ZFHX3 has also been found to be closely related to neuronal and myogenic differentiation in brain and heart (36–38). In cell signaling, ZFHX3 interacts with a protein that specifically inhibits signal transducer and activator of transcription 3 (STAT3). ZFHX3 can activate this protein inhibitor of activated STAT 3 (PIAS3) as well. Research shows that in AF models, tachycardia induced a decreased expression of ZFHX3, then STAT3 signaling is activated by decreasing PIAS3 activity. Therefore, the inflammatory process of atrial tissue resulting from the down-regulation of ZFHX3 may influence the occurrence of atrial arrhythmia (39). These lines of evidence provide a mechanistic basis for the genetic association between ZFHX3 and AF. Though we did not find the causal association between rs2106261 and VHD directly, they all involved in the same biological mechanisms such as inflammatory mechanisms, which may play a significant role in the causal effect of AF on VHD.

An observational study performed by Davutoglu et al. (40) reported that the pathogenesis of VHD was also associated with persistent serum inflammatory mediators that were strongly linked to the severity of valve involvement, valve scarring, subsequent valve calcification, and reduced functional status. Cho et al. (41) identified that VHD was no longer thought to be a simple passive process caused by calcium deposition that occurs with advanced age, immunological and inflammatory responses were also involved, including oxidized lipids, various cytokines, and biomineralization. From a clinical perspective, several studies have suggested that AF can lead to atrial dilation, atrial systolic dysfunction and reconstruction of atrioventricular valve ring, which may cause damage to the valve, but it would be improved if sinus rhythm was restored (42–45). However, no studies have shown whether it is affected by only one mechanism or multiple mechanisms, which needs to be further confirmed.

A chief strength of the present study is that we assessed the causal associations between AF and VHD in the same study population using the MR method. Because alleles are randomly combined and fixed at conception, these genetic variations are not affected by socioeconomic status and associated attributes, or subsequent disease. It means that our results from the MR method are more accurate than from observational studies, and represent a lifetime risk of VHD on AF. Another strength is that VHD GWAS of our study was finished just in European ancestry populations, which could reduce bias due to population stratification.

Three limitations should be acknowledged. Firstly, since no specific diseases such as mitral regurgitation were found in GWAS database, we only analyzed AF and VHD without subgroup analysis. Secondly, there is no way to obtain information about disease severity or classification because we only extract data from disease diagnosis, not from examination indicators such as cardiac ultrasound. Thirdly, it has been well accepted that the risk factors are various in different races and ethnicities (46). Therefore, the reliability of the causal associations should be validated in other races.

## Conclusion

In conclusion, our results provide strong evidence of a causal relationship between AF and VHD by using MR method. Based on this result, we recommend that early treatment should be performed for AF patients to reduce the risk of VHD.

## Data Availability Statement

The datasets presented in this study can be found in online repositories. The names of the repository/repositories and accession number(s) can be found in the article/Supplementary Material.

## Ethics Statement

Ethical approval was not provided for this study on human participants because in this study, we obtained the summary data from published studies, which have already been approved by institutional review committees in their respective studies. Therefore, no further sanction was required. The patients/participants provided their written informed consent to participate in this study.

## Author Contributions

JG and HJ designed the research. JG and YB performed the research, analyzed the data, and wrote the paper. All authors contributed to the article and approved the submitted version.

## Funding

The work was supported by grants from the Chinese Academy of Medical Sciences (CAMS) Innovation Fund for Medical Sciences (CIFMS) (ID 2016-I2M-3-024). The funding source had no role in the design and conduct of the study, collection, management, analysis, and interpretation of the data; preparation, review, or approval of the manuscript, and the decision to submit the manuscript for publication.

## Conflict of Interest

The authors declare that the research was conducted in the absence of any commercial or financial relationships that could be construed as a potential conflict of interest.

## Publisher's Note

All claims expressed in this article are solely those of the authors and do not necessarily represent those of their affiliated organizations, or those of the publisher, the editors and the reviewers. Any product that may be evaluated in this article, or claim that may be made by its manufacturer, is not guaranteed or endorsed by the publisher.

## Supplementary Material

Supplementary Figure 1 Forest plot to visualize causal effect of each single SNP on total VHD risk. **(A)** VHD including rheumatic fever. **(B)** non-rheumatic VHD. MR, Mendelian randomization; VHD, Valvular heart disease.

Supplementary Figure 2 Leave-one-out plot to visualize causal effect of AF on total VHD risk when leaving one SNP out. **(A)** VHD including rheumatic fever. **(B)** non-rheumatic VHD. MR, Mendelian randomization.

Supplementary Table 1 List of genetic instruments for AF and log odds ratios of VHD risk by each instrumental SNPs (GWAS significance with p <5 × 10-8 and linkage disequilibrium threshold with r2 <0.005).
