# Supplementary figures and images for: Genetically Predicted Atrial Fibrillation and Valvular Heart Disease: A Two-Sample Mendelian Randomization Study
Source: Front Cardiovasc Med. 2022 Mar 28;9:845734. doi: 10.3389/fcvm.2022.845734 (PMC8996053; doi:10.3389/fcvm.2022.845734)

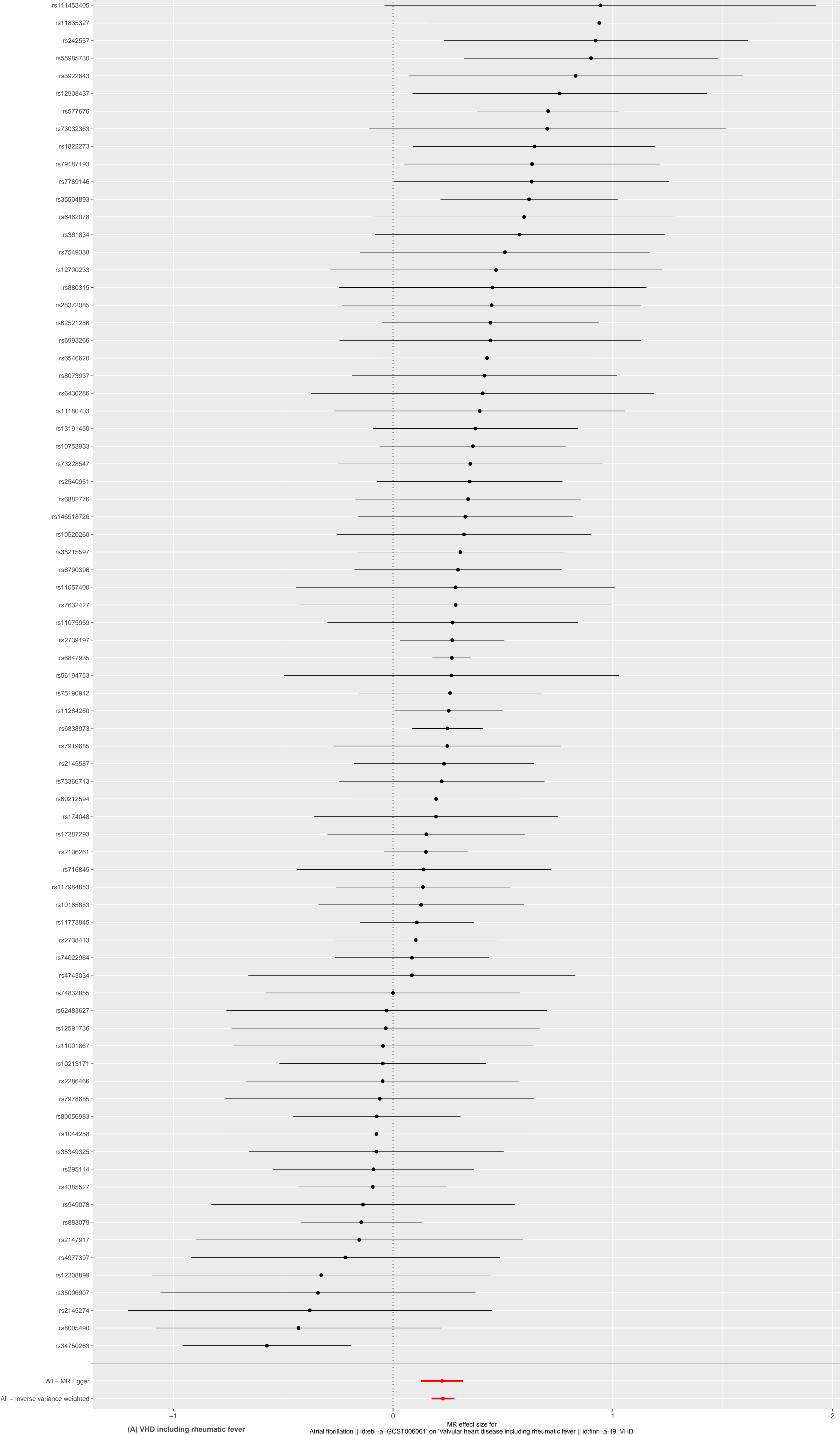

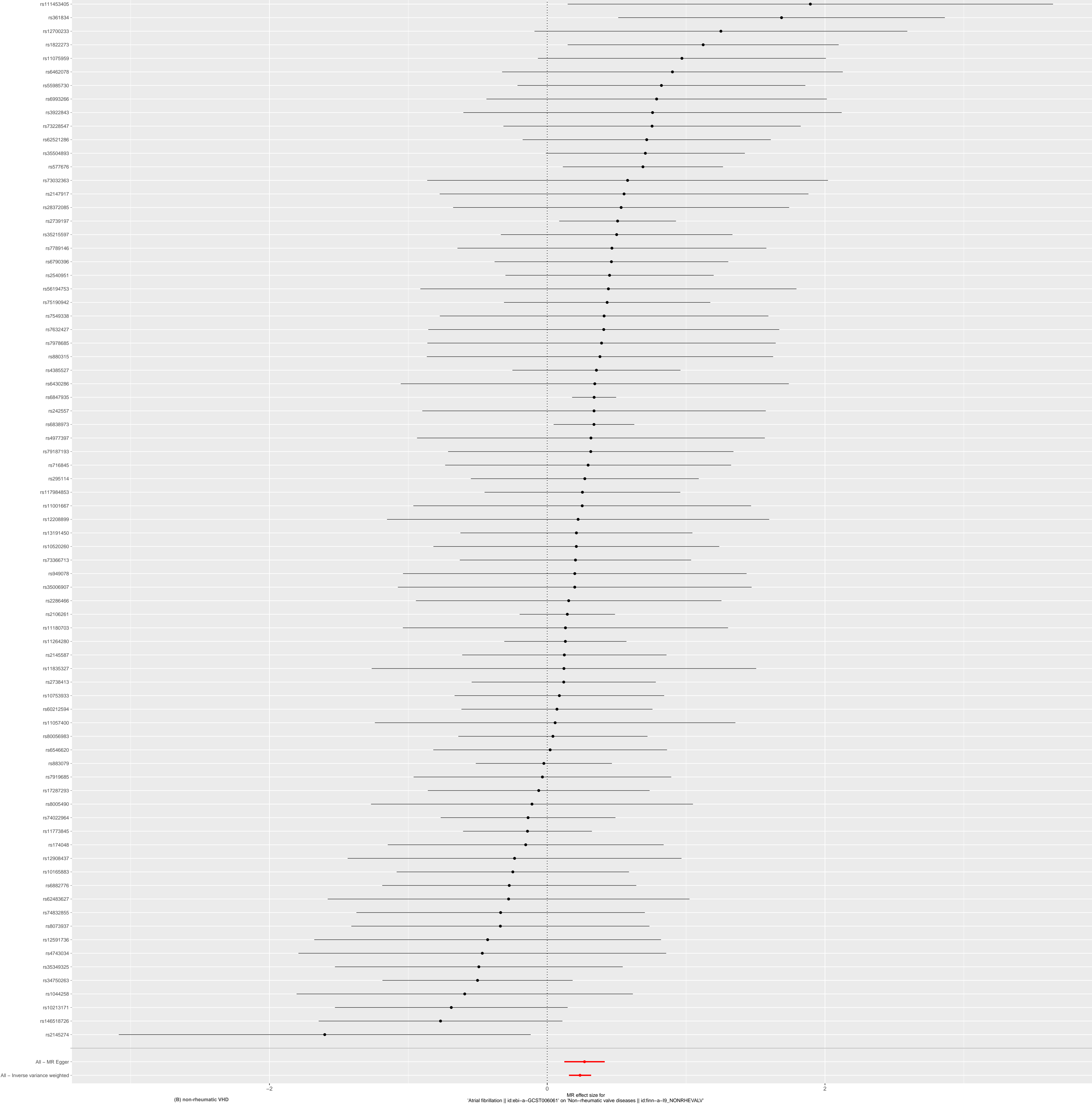

Supplement: Supplementary Figure 1 — Forest plot to visualize causal effect of each single SNP on total VHD risk. (A) VHD including rheumatic fever. (B) non-rheumatic VHD. MR, Mendelian randomization; VHD, Valvular heart disease. [file Data_Sheet_1.PDF]

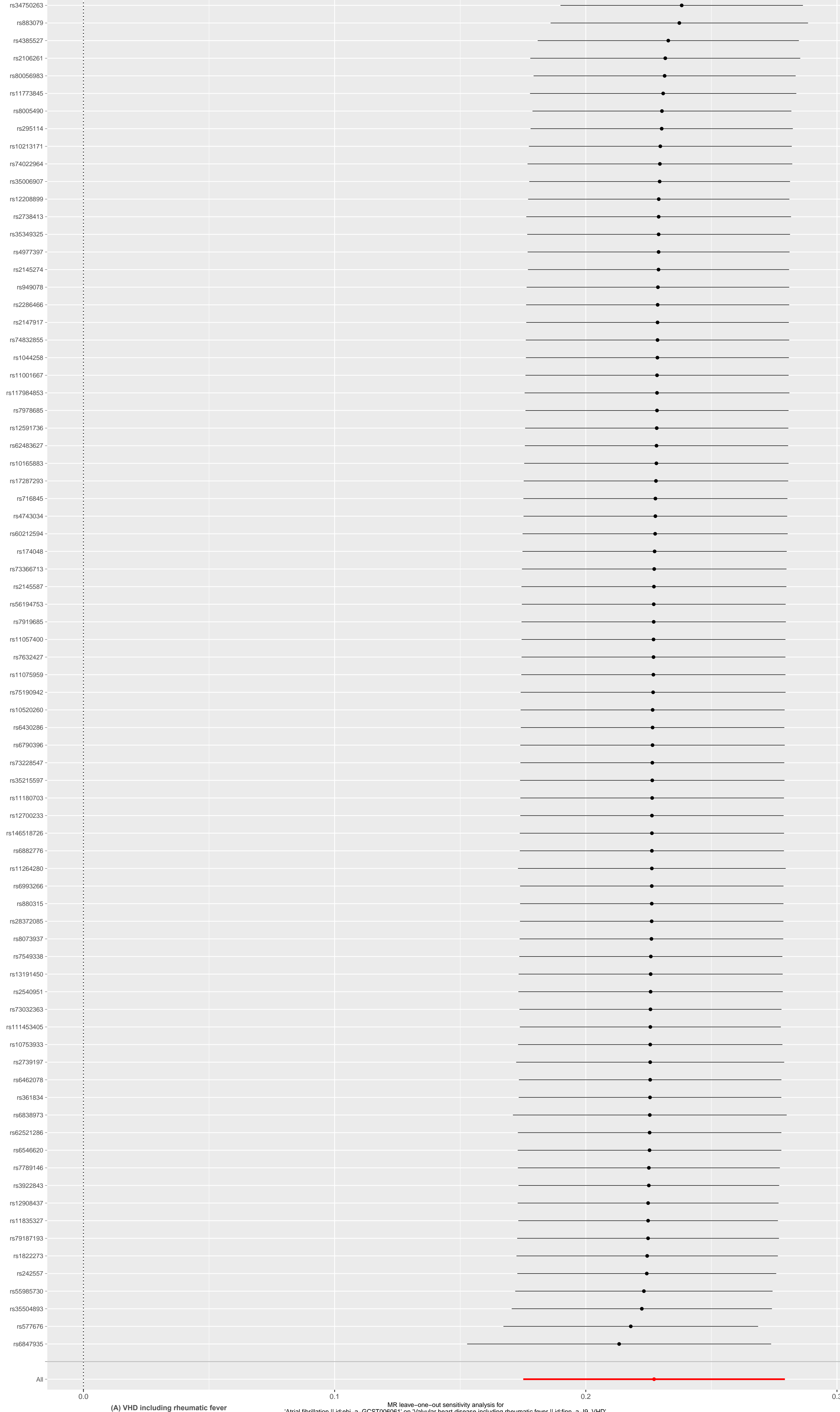

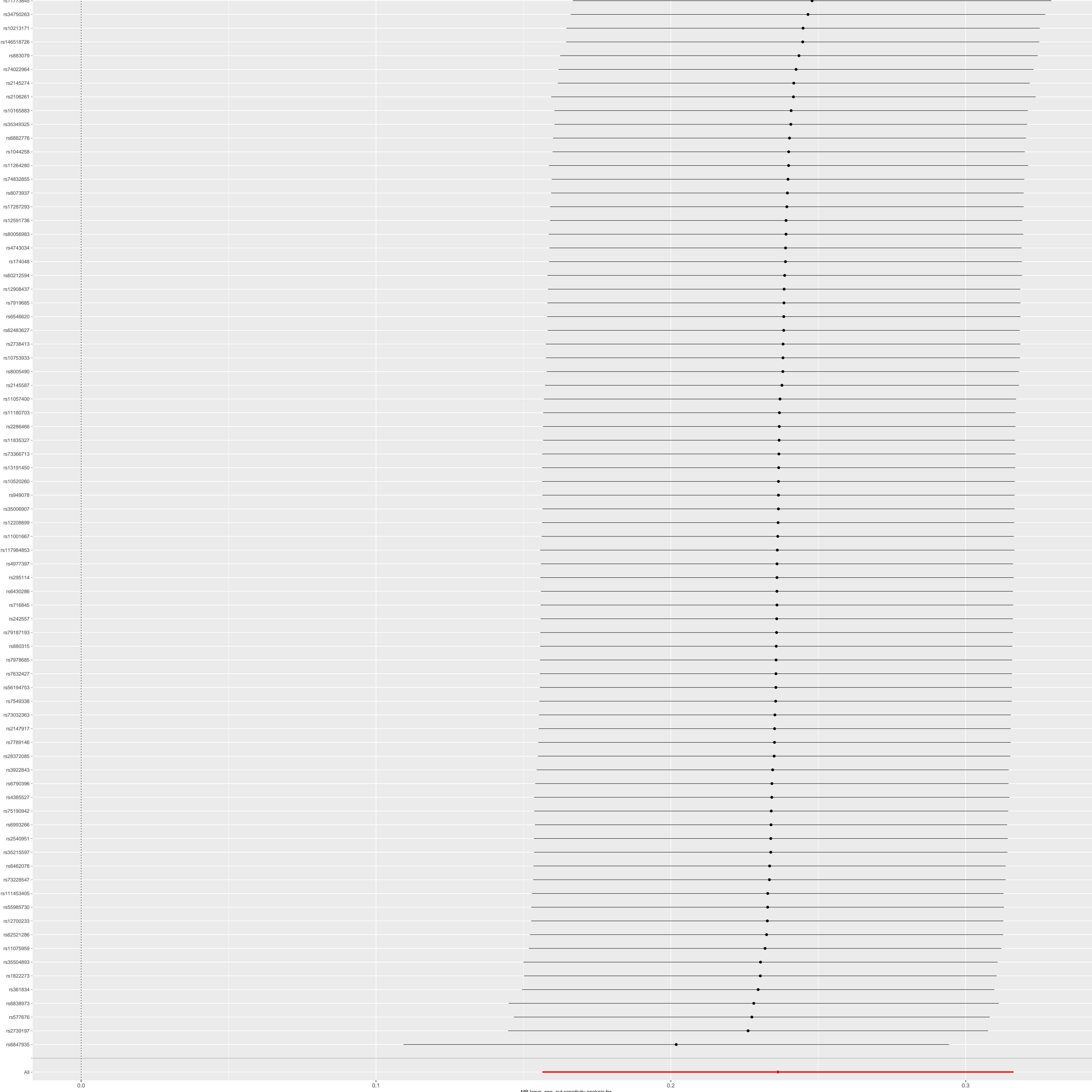

Supplement: Supplementary Figure 2 — Leave-one-out plot to visualize causal effect of AF on total VHD risk when leaving one SNP out. (A) VHD including rheumatic fever. (B) non-rheumatic VHD. MR, Mendelian randomization. [file Data_Sheet_2.PDF]
