# Supplementary material for: Genetically Predicted Atrial Fibrillation and Valvular Heart Disease: A Two-Sample Mendelian Randomization Study
Source: Front Cardiovasc Med. 2022 Mar 28;9:845734. doi: 10.3389/fcvm.2022.845734 (PMC8996053; doi:10.3389/fcvm.2022.845734)
Supplement: Supplementary Table 1 — List of genetic instruments for AF and log odds ratios of VHD risk by each instrumental SNPs (GWAS significance with p <5 × 10^-8^ and linkage disequilibrium threshold with r2 <0.005). [file Table_1.DOCX]

**Supplementary Table S1** List of genetic instruments for AF and log odds ratios of VHD risk by each instrumental SNPs (GWAS significance with p <5×10^-8^ and linkage disequilibrium threshold with r^2^ <0.005).

| No. | SNP | Gene | Chr. | EA | OA | EAF.VHD | Beta.AF | SE.AF | Beta.VHD | SE.VHD |
| --- | --- | --- | --- | --- | --- | --- | --- | --- | --- | --- |
| 1 | rs10165883 | - | 2 | T | C | 0.4889 | -0.0642 | 0.0072 | -0.0082 | 0.0153 |
| 2 | rs10213171 | ARHGAP10 | 4 | G | C | 0.8948 | 0.1041 | 0.0139 | -0.0048 | 0.025 |
| 3 | rs1044258 | ARMH3/LOC107984029 | 10 | C | T | 0.3523 | -0.0463 | 0.0076 | 0.0035 | 0.016 |
| 4 | rs10520260 | HAND2 | 4 | G | A | 0.3771 | -0.0539 | 0.0079 | -0.0174 | 0.0159 |
| 5 | rs10753933 | PPFIA4 | 1 | T | G | 0.3549 | -0.0743 | 0.0072 | 0.027 | 0.0161 |
| 6 | rs11001667 | LRMDA | 10 | G | A | 0.1495 | 0.0619 | 0.0091 | -0.0028 | 0.0215 |
| 7 | rs11057400 | CCDC92 | 12 | T | C | 0.2924 | -0.0456 | 0.0077 | -0.013 | 0.0169 |
| 8 | rs11075959 | ZFHX3 | 16 | G | A | 0.9621 | 0.1397 | 0.0216 | 0.0379 | 0.0406 |
| 9 | rs111453405 | PKP2 | 12 | C | T | 0.93611 | 0.0627 | 0.0111 | -0.0591 | 0.0314 |
| 10 | rs11180703 | LOC105369844 | 12 | A | G | 0.484 | -0.0457 | 0.0073 | -0.018 | 0.0154 |
| 11 | rs11264280 | - | 1 | C | T | 0.6466 | 0.127 | 0.0078 | -0.0322 | 0.016 |
| 12 | rs11773845 | CAV1 | 7 | C | A | 0.4397 | 0.1162 | 0.0072 | -0.0126 | 0.0154 |
| 13 | rs117984853 | - | 6 | G | T | 0.8696 | 0.1132 | 0.0136 | -0.0154 | 0.0229 |
| 14 | rs11835327 | LOC105369998 | 12 | G | A | 0.08881 | 0.0681 | 0.0122 | 0.0639 | 0.0269 |
| 15 | rs12208899 | - | 6 | A | G | 0.8037 | 0.049 | 0.0087 | -0.016 | 0.0193 |
| 16 | rs12591736 | - | 15 | A | G | 0.1475 | -0.0606 | 0.0102 | 0.002 | 0.0217 |
| 17 | rs12700233 | SUN1 | 7 | G | T | 0.5714 | 0.0403 | 0.0074 | -0.0189 | 0.0155 |
| 18 | rs12908437 | IGF1R | 15 | T | C | 0.3517 | -0.0468 | 0.0073 | 0.0355 | 0.016 |
| 19 | rs13191450 | - | 6 | C | A | 0.293 | -0.0704 | 0.0075 | -0.0264 | 0.0168 |
| 20 | rs146518726 | - | 1 | G | A | 0.96297 | 0.1617 | 0.0254 | -0.0532 | 0.0403 |
| 21 | rs17287293 | LOC105369698 | 12 | G | A | 0.107 | -0.108 | 0.0103 | -0.0164 | 0.0248 |
| 22 | rs174048 | - | 5 | T | C | 0.7902 | 0.0665 | 0.0098 | -0.013 | 0.0188 |
| 23 | rs1822273 | NAV2 | 11 | G | A | 0.1995 | -0.0683 | 0.0082 | 0.0439 | 0.0192 |
| 24 | rs2106261 | ZFHX3 | 16 | T | C | 0.2252 | 0.1872 | 0.0088 | 0.028 | 0.0183 |
| 25 | rs2145274 | - | 20 | A | C | 0.96599 | -0.1015 | 0.0141 | -0.0384 | 0.0429 |
| 26 | rs2145587 | AKAP6 | 14 | A | G | 0.3737 | 0.0754 | 0.0079 | 0.0175 | 0.0158 |
| 27 | rs2147917 | ZNF292 | 6 | C | T | 0.4907 | -0.0403 | 0.007 | -0.0062 | 0.0153 |
| 28 | rs2286466 | RPS2/SNORA64/ SNORA10/SNORA78/SNHG9 | 16 | G | A | 0.8678 | 0.0718 | 0.0095 | -0.0034 | 0.0228 |
| 29 | rs242557 | MAPT | 17 | A | G | 0.4679 | -0.0439 | 0.0075 | -0.0405 | 0.0155 |
| 30 | rs2540951 | LINC02576/LOC107984063 | 2 | G | A | 0.3293 | -0.0753 | 0.0073 | -0.0263 | 0.0162 |
| 31 | rs2738413 | SYNE2 | 14 | G | A | 0.5222 | -0.0807 | 0.0072 | -0.0083 | 0.0153 |
| 32 | rs2739197 | PITX2 | 4 | G | C | 0.296 | 0.1389 | 0.0085 | 0.0374 | 0.0168 |
| 33 | rs28372085 | XPO7 | 8 | T | C | 0.05946 | -0.093 | 0.0119 | -0.0417 | 0.0323 |
| 34 | rs295114 | SPATS2L | 2 | C | T | 0.6351 | -0.0676 | 0.0073 | -0.006 | 0.0158 |
| 35 | rs34750263 | - | 5 | T | C | 0.7195 | 0.0873 | 0.0076 | -0.0501 | 0.0171 |
| 36 | rs35006907 | LOC105375743 | 8 | C | A | 0.6907 | 0.0454 | 0.0076 | 0.0155 | 0.0166 |
| 37 | rs35215597 | WIPF1 | 2 | A | G | 0.7717 | -0.0764 | 0.0086 | 0.0234 | 0.0183 |
| 38 | rs35349325 | - | 12 | C | T | 0.435 | -0.0524 | 0.0073 | 0.004 | 0.0155 |
| 39 | rs35504893 | TTN/TTN-AS1 | 2 | C | T | 0.7824 | 0.09 | 0.0087 | -0.0557 | 0.0185 |
| 40 | rs361834 | TUBA8 | 22 | A | G | 0.3906 | -0.047 | 0.0075 | -0.0271 | 0.0158 |
| 41 | rs3922843 | SCN5A | 3 | A | G | 0.2281 | -0.0472 | 0.0082 | 0.0392 | 0.0183 |
| 42 | rs4385527 | AOPEP | 9 | A | G | 0.3529 | 0.092 | 0.0073 | -0.0085 | 0.0159 |
| 43 | rs4743034 | ZNF462 | 9 | A | G | 0.2175 | 0.049 | 0.0083 | 0.0042 | 0.0186 |
| 44 | rs4977397 | SLC24A2 | 9 | G | A | 0.4291 | -0.0432 | 0.0075 | 0.0094 | 0.0155 |
| 45 | rs55985730 | OPN1SW | 7 | G | T | 0.08273 | 0.0957 | 0.017 | 0.0862 | 0.0282 |
| 46 | rs56194753 | - | 2 | A | G | 0.91655 | -0.0715 | 0.0114 | 0.019 | 0.0278 |
| 47 | rs577676 | - | 1 | C | T | 0.6185 | -0.0962 | 0.0072 | 0.0679 | 0.0159 |
| 48 | rs60212594 | SYNPO2L/SYNPO2L-AS1 | 10 | C | G | 0.1452 | -0.1097 | 0.0102 | -0.0215 | 0.0216 |
| 49 | rs62483627 | COG5 | 7 | G | A | 0.7709 | 0.0489 | 0.0084 | 0.0014 | 0.0182 |
| 50 | rs62521286 | FBXO32 | 8 | A | G | 0.93388 | 0.1224 | 0.0148 | -0.0542 | 0.0309 |
| 51 | rs6430286 | MBD5 | 2 | A | G | 0.391 | 0.0397 | 0.0071 | 0.0162 | 0.0158 |
| 52 | rs6462078 | CREB5 | 7 | A | C | 0.8318 | 0.058 | 0.0086 | 0.0346 | 0.0204 |
| 53 | rs6546620 | KIF3C | 2 | C | T | 0.7124 | 0.0708 | 0.0093 | 0.0303 | 0.0171 |
| 54 | rs6790396 | SCN10A | 3 | C | G | 0.4694 | 0.0636 | 0.0073 | -0.0188 | 0.0153 |
| 55 | rs6838973 | - | 4 | C | T | 0.4991 | -0.1842 | 0.0072 | 0.0457 | 0.0153 |
| 56 | rs6847935 | - | 4 | T | A | 0.2357 | 0.4029 | 0.0082 | 0.1077 | 0.0181 |
| 57 | rs6882776 | NKX2-5 | 5 | G | A | 0.5959 | -0.06 | 0.0079 | 0.0205 | 0.0157 |
| 58 | rs6993266 | PTK2 | 8 | G | A | 0.4184 | 0.0443 | 0.0072 | -0.0196 | 0.0155 |
| 59 | rs716845 | KCNN2 | 5 | G | A | 0.7456 | 0.0594 | 0.008 | -0.0083 | 0.0175 |
| 60 | rs73032363 | THRB | 3 | A | G | 0.755 | -0.0432 | 0.0078 | 0.0303 | 0.0179 |
| 61 | rs73228547 | PHLDB2 | 3 | G | A | 0.8479 | -0.0694 | 0.0106 | 0.0244 | 0.0213 |
| 62 | rs73366713 | ATXN1 | 6 | A | G | 0.1044 | -0.1052 | 0.0112 | -0.0233 | 0.0251 |
| 63 | rs74022964 | - | 15 | T | C | 0.2057 | 0.1059 | 0.0097 | 0.0091 | 0.019 |
| 64 | rs74832855 | LINC01681 | 1 | G | A | 0.03463 | 0.1425 | 0.0199 | 0 | 0.042 |
| 65 | rs75190942 | KCNJ5 | 11 | A | C | 0.09648 | 0.1256 | 0.0136 | 0.0326 | 0.0265 |
| 66 | rs7549338 | IL6R | 1 | C | G | 0.5235 | -0.0454 | 0.0071 | 0.0231 | 0.0153 |
| 67 | rs7632427 | - | 3 | T | C | 0.5053 | -0.0425 | 0.0074 | 0.0121 | 0.0154 |
| 68 | rs7789146 | KCNH2 | 7 | G | A | 0.7672 | -0.0571 | 0.0092 | 0.036 | 0.0182 |
| 69 | rs79187193 | LOC102723321 | 1 | G | A | 0.94412 | -0.1116 | 0.0182 | 0.0706 | 0.0332 |
| 70 | rs7919685 | REEP3 | 10 | G | T | 0.5158 | -0.0579 | 0.0071 | 0.0143 | 0.0153 |
| 71 | rs7978685 | - | 12 | T | C | 0.8148 | -0.0547 | 0.0079 | -0.0033 | 0.0196 |
| 72 | rs8005490 | CFL2 | 14 | T | C | 0.4457 | -0.0465 | 0.0073 | -0.02 | 0.0154 |
| 73 | rs80056983 | SH3PXD2A/SH3PXD2A-AS1 | 10 | T | C | 0.1215 | 0.122 | 0.0102 | -0.009 | 0.0237 |
| 74 | rs8073937 | - | 17 | G | A | 0.4357 | -0.0504 | 0.0074 | 0.021 | 0.0155 |
| 75 | rs880315 | CASZ1 | 1 | C | T | 0.4117 | 0.0437 | 0.0075 | 0.0198 | 0.0156 |
| 76 | rs883079 | TBX5 | 12 | C | T | 0.7047 | 0.1196 | 0.0079 | 0.0173 | 0.0168 |
| 77 | rs949078 | - | 11 | C | T | 0.21 | -0.0534 | 0.0081 | -0.0073 | 0.0188 |

Chr. indicates chromosome; EA, effect allele; OA, other allele; EAF, effect allele frequency.
